# G protein–receptor kinases 5/6 are the key regulators of G protein–coupled receptor 35–arrestin interactions

**DOI:** 10.1016/j.jbc.2023.105218

**Published:** 2023-09-01

**Authors:** Amlan Ganguly, Tezz Quon, Laura Jenkins, Babu Joseph, Rima Al-awar, Andy Chevigne, Andrew B. Tobin, David E. Uehling, Carsten Hoffmann, Julia Drube, Graeme Milligan

**Affiliations:** 1 Centre for Translational Pharmacology, School of Molecular Biosciences, Advanced Research Centre (ARC), College of Medical, Veterinary and Life Sciences University of Glasgow, Glasgow, UK; 2 Drug Discovery Program, Ontario Institute for Cancer Research, MaRS Centre, Toronto, Ontario, Canada; 3 Division of Immuno-Pharmacology and Interactomics, Department of Infection and Immunity, Luxembourg Institute of Health, Esch-sur-Alzette, Luxembourg; 4 Institute for Molecular Cell Biology, CMB—Center for Molecular Biomedicine, University Hospital Jena, Friedrich Schiller University Jena, Jena, Germany

**Keywords:** GPR35, G protein–coupled receptor kinases, arrestins, phosphorylation, phospho-site–specific antisera

## Abstract

Human G protein–coupled receptor 35 is regulated by agonist-mediated phosphorylation of a set of five phospho-acceptor amino acids within its C-terminal tail. Alteration of both Ser^300^ and Ser^303^ to alanine in the GPR35a isoform greatly reduces the ability of receptor agonists to promote interactions with arrestin adapter proteins. Here, we have integrated the use of cell lines genome edited to lack expression of combinations of G protein receptor kinases (GRKs), selective small molecule inhibitors of subsets of these kinases, and antisera able to specifically identify either human GPR35a or mouse GPR35 only when Ser^300^ and Ser^303^ (orce; the equivalent residues in mouse GPR35) have become phosphorylated to demonstrate that GRK5 and GRK6 cause agonist-dependent phosphorylation of these residues. Extensions of these studies demonstrated the importance of the GRK5/6-mediated phosphorylation of these amino acids for agonist-induced internalization of the receptor. Homology and predictive modeling of the interaction of human GPR35 with GRKs showed that the N terminus of GRK5 is likely to dock in the same methionine pocket on the intracellular face of GPR35 as the C terminus of the α5 helix of Gα_13_ and, that while this is also the case for GRK6, GRK2 and GRK3 are unable to do so effectively. These studies provide unique and wide-ranging insights into modes of regulation of GPR35, a receptor that is currently attracting considerable interest as a novel therapeutic target in diseases including ulcerative colitis.

Although it is well known that members of the G protein–coupled receptor kinase (GRK) family play key roles in the phosphorylation and regulation of many G protein–coupled receptors (GPCRs), the specific contribution of individual GRKs, of which GRK2, GRK3, GRK5 and GRK6 are widely expressed, has often been poorly explored and defined (1, 2, 3). This has reflected a combination of the routine coexpression of multiple GRK isoforms and the limited range of well-characterized small molecule inhibitors of individual GRKs or of subsets of the larger group. Key challenges lie in the precise determination of sites of phosphorylation and linking this to the activity of specific GRKs, or other protein kinases, and the regulation of defined cellular responses. We have addressed this challenge previously by employing mass spectrometry–based phospho-proteomics, allowing for the rational design of receptor phospho-site–specific antibodies (4, 5, 6). Using such antibodies as tools to probe the phosphorylation status of receptors, we have determined that the pattern of phosphorylation of any given GPCR can be dependent on the cell type in which the receptor is expressed (7) and that this pattern, or phosphorylation barcode (8, 9, 10), is one mechanism that can contribute to cell type–specific receptor signaling. To fully appreciate the regulation of receptor activity, it is hence vital that precise sites of basal and regulated phosphorylation are defined and that the specific protein kinases that promote each receptor-phosphorylation event are delineated.

One area in which this knowledge is generally lacking is for receptors which have, until now, been understudied but for which there is an emerging interest in their potential as novel targets for the treatment of human disease. One such receptor is GPR35, which is currently attracting considerable interest as a therapeutic target in areas ranging from lower gut inflammation to nonalcoholic steatohepatitis (11, 12). The sites of agonist-induced phosphorylation in GPR35 cluster within the relatively short, intracellular C-terminal tail and have been mapped by combinations of mass spectrometry, [^32^P] labeling and mutagenesis (5). These studies showed that phosphorylation of Ser^300^ and Ser^303^ in human GPR35a, and of the equivalent residues in the mouse and rat orthologues, were key to effective interactions of the receptor with arrestin-3. Moreover, phosphorylation of these residues occurred in an almost entirely agonist-dependent manner. Herein, we build on these studies by using combinations of GRK subtype KO cell lines (13) and reconstitution of function with individual GRKs, as well as a pSer^300^-pSer^303^ human GPR35a directed antiserum (5) and a group of selective small molecule GRK inhibitors (14, 15). Our studies show that GRK5 and GRK6 are the key mediators of the phosphorylation of Ser^300^ and Ser^303^ in human GPR35a, as well as the equivalent residues in mouse GPR35. This defines effective interactions of this receptor with arrestins, a process directly associated with receptor internalization.

## Results

### Agonist-induced interactions of hGPR35a and arrestins require expression of one or more GRK

Human embryonic kidney (HEK) 293 cells were transiently transfected to coexpress human (h) GPR35a, tagged at the C terminus with enhanced yellow fluorescent protein (eYFP) (hGPR35a-eYFP), and arrestin-3 tagged with *Renilla* luciferase (arrestin-3-RLuc). In the presence of a luciferase substrate addition of the GPR35 agonist lodoxamide, that displays high potency at human GPR35 (16), resulted in a concentration-dependent increase in measured bioluminescence resonance energy transfer (BRET) (Fig. 1) with pEC_50_ 8.04 ± 0.22 (mean ± SD, n = 3). Equivalent studies using a previously characterized C-terminal phospho-acceptor site–deficient mutant (PDM) of hGPR35a (hGPR35a-PDM-eYFP) (5) in which all five of the serine/threonine residues within the receptor C-terminal tail had been altered to alanines resulted in no detectable increase in BRET signal (Fig. 1). Effective interactions between the receptor and arrestin-3 in response to lodoxamide required the presence of one or more members of the GRK family. This was evident because when such studies were performed in a clone of HEK293 cells genetically engineered to lack all four of the widely expressed GRK isoforms (ΔGRK2/3/5/6) (13) then, even when employing WT hGPR35a-eYFP, only a very minor response to lodoxamide was observed (Fig. 1). As anticipated from the above, no response to lodoxamide was observed when hGPR35a-PDM-eYFP and arrestin-3-RLuc were cointroduced into ΔGRK2/3/5/6293 cells (Fig. 1). Similar outcomes (lodoxamide pEC_50_ 7.71 ± 0.10, mean ± SD, n = 3 for WT) were produced when arrestin-3-RLuc was replaced with arrestin-2-Rluc (Fig. S1).

**Figure 1:**
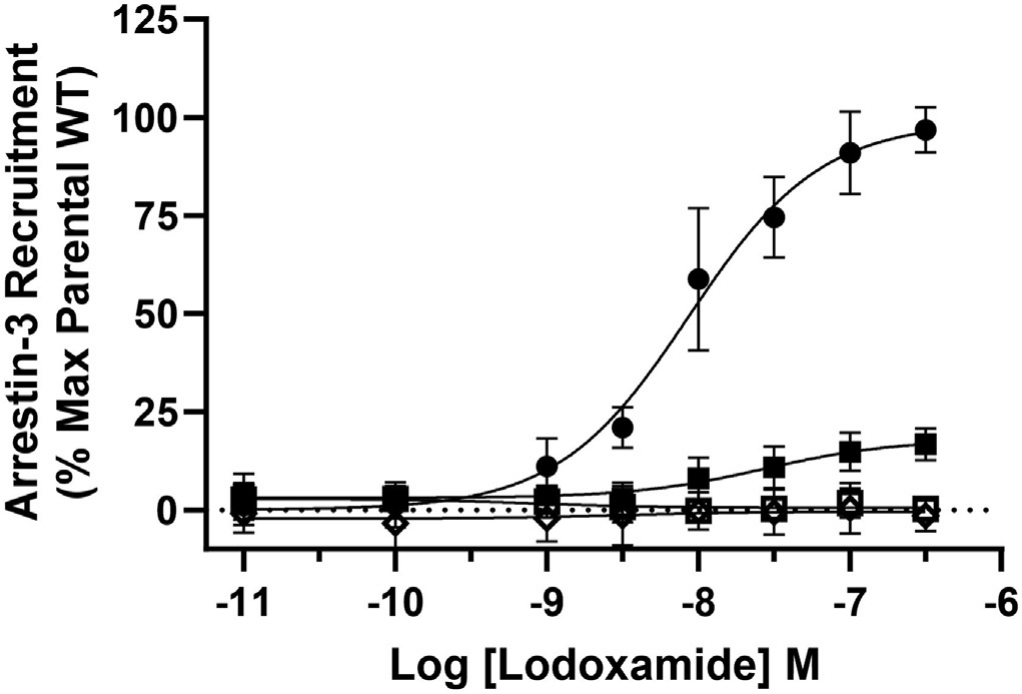
**Agonist-induced interactions between human GPR35a and arrestin-3 require the presence of one or more GRK isoforms.** Parental HEK293 cells (*filled circles, open diamonds*) or a clone genome edited to lack expression of each of GRK2, GRK3, GRK5, and GRK6 (ΔGRK2/3/5/6) (*filled squares, open squares*) were transfected transiently to express either hGPR35a-eYFP (*filled symbols*) or hGPR35a-PDM-eYFP (*open symbols*) and arrestin-3-RLuc. Cells were exposed to the indicated concentrations of lodoxamide for 5 min and after substrate addition BRET was measured. Data are the mean ± SD of outcomes from three independent experiments. BRET, bioluminescence resonance energy transfer; GRK, G protein–coupled receptor kinase; HEK, Human embryonic kidney.

GRK2 and GRK3 form a subfamily of the widely expressed GRKs, as do GRK5 and GRK6 (2, 17). To define which GRK isoform(s) might promote agonist-induced hGPR35a/arrestin-3 interactions, we initially used a combination of HEK293-derived cell lines engineered to lack expression of either GRK2 and GRK3 (ΔGRK2/3) or GRK5 and GRK6 (ΔGRK5/6) (13), alongside selective small molecule inhibitors. Introduction of hGPR35a-eYFP and arrestin-3-RLuc into ΔGRK5/6 cells did not result in lodoxamide-induced BRET and, indeed, phenocopied the observations made in ΔGRK2/3/5/6 cells (Fig. 2*A*). By contrast, after cotransfection of hGPR35a-eYFP and arrestin-3-RLuc into ΔGRK2/3 cells, addition of lodoxamide resulted in BRET signals (Fig. 2*A*) very similar in potency (pEC_50_ 8.09 ± 0.04, mean ± SD, n = 3) and, indeed, slightly larger in magnitude, than those in parental HEK293 cells.

**Figure 2:**
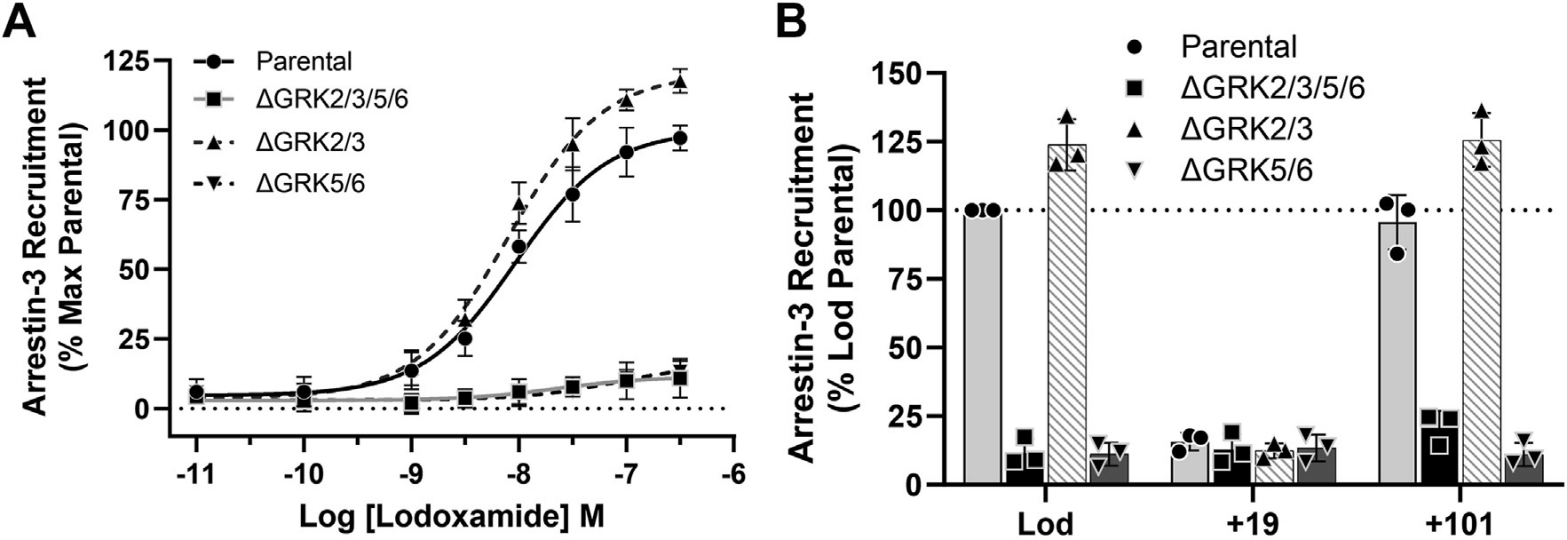
**GRK5 and/or GRK6 but not GRK2 and/or GRK3 are essential to allow agonist-promoted human GPR35a–arrestin-3 interactions.** Studies akin to Figure 1 were performed after transient coexpression of hGPR35a-eYFP and arrestin-3-RLuc into each of parental HEK293 cells (*circles*) or clones genome edited to lack expression of GRK2 and GRK3 (ΔGRK2/3) (*triangles*), GRK5 and GRK6 (ΔGRK5/6) (inverted *triangles*), or all four of these GRKs (ΔGRK 2/3/5/6) (*squares*). *A,* cells were exposed to the indicated concentrations of lodoxamide for 5 min and after substrate addition BRET was measured. Data are the mean ± SD of outcomes from three independent experiments. *B,* cells were exposed to 100 nM lodoxamide for 5 min after pre-exposure or not to either compound 101 or compound 19 (each at 10 μM) for 30 min. Data show individual outcomes from three independent studies. BRET, bioluminescence resonance energy transfer; GRK, G protein–coupled receptor kinase; HEK, Human embryonic kidney; RLuc, *Renilla* luciferase.

Compound 101 (3-[[[4-methyl-5-(4-pyridyl)-4H-1,2,4-triazole-3-yl] methyl] amino]-N-[2-(trifluoromethyl) benzyl] benzydaminehydrochloride) is a well-studied, small molecule GRK2/3 selective inhibitor (18, 19). Both in WT and in ΔGRK2/3 cells preaddition of compound 101 (10 μM) did not reduce the capacity of lodoxamide to promote hGPR35a-eYFP–arrestin-3-RLuc interactions (Fig. 2*B*). GRK5/6 inhibitors have not been as widely developed and studied. However, “compound 19” ((*S*)-*N*2-(1-(5-chloropyridin-2-yl)ethyl)-*N*4-(5-ethyl-1*H*-pyrazol-3-yl)-5-methoxyquinazoline-2,4-diamine) (10 μM) reported as one of a series of GRK5/6-selective inhibitors (14, 15) was able to fully prevent lodoxamide-mediated hGPR35a-eYFP–arrestin-3-RLuc interactions in both parental and ΔGRK2/3293 cells (Fig. 2*B*). Additional compounds from this series, including compounds 15, 16, 17 (14), were able to replicate this effect (Fig. S2).

### Expression of GRK5 or GRK6 is sufficient to allow hGPR35a–arrestin-3 interactions

To extend the analysis to individual GRK isoforms, we transiently introduced hGPR35a-eYFP and arrestin-3-RLuc into ΔGRK2/3/5/6293 cells alongside plasmids able to express each of GRK2, GRK3, GRK5, and GRK6. Here, introduction of either GRK5 or GRK6 was able to largely reconstitute lodoxamide-induced hGPR35a-eYFP/arrestin-3 interactions. By contrast, introduction of either GRK2 or GRK3 had much less pronounced effects (Fig. 3). To assess if the variation of extent of reconstitution could reflect intrinsic differences in the actions of the different GRKs or might simply reflect transfection and expression level variation between them, we initially took advantage of the fact that each of the introduced GRK isoforms was a fused to the large BiT (LgBiT) fragment (20) of the NanoBiT complementation technology (21, 22). This allowed us to employ an anti-LgBiT mAb to detect the relative levels of each GRK (Fig. 4). Following transfection of either parental HEK293 (Fig. 4*A*) or ΔGRK2/3/5/6293 (Fig. 4*B*) cells, relatively similar levels of each GRK was detected. Similar immunoblots, but now performed using GRK isoform–directed antibodies, confirmed successful expression of each isoform, with a degree of previously reported (23) cross-reactivity with GRK5 for the nominally GRK6 directed antibody in both ΔGRK2/3/5/6 (Fig. S3*A*) and parental (Fig. S3*B*) 293 cells. The additional 18 kDa molecular mass provided by the fusion of the GRKs with the LgBiT sequence also allowed immunoblots with the GRK-isoform antibodies to concurrently codetect the relative levels of the introduced forms of GRK-LgBiT species and endogenous levels of each of the GRKs in the parental HEK293 cells (Fig. S3*B*). Such studies showed that the levels of introduced GRKs were similar to (GRK3, GRK6), or rather higher (GRK2, GRK5), than endogenous levels in the parental cells.

**Figure 3:**
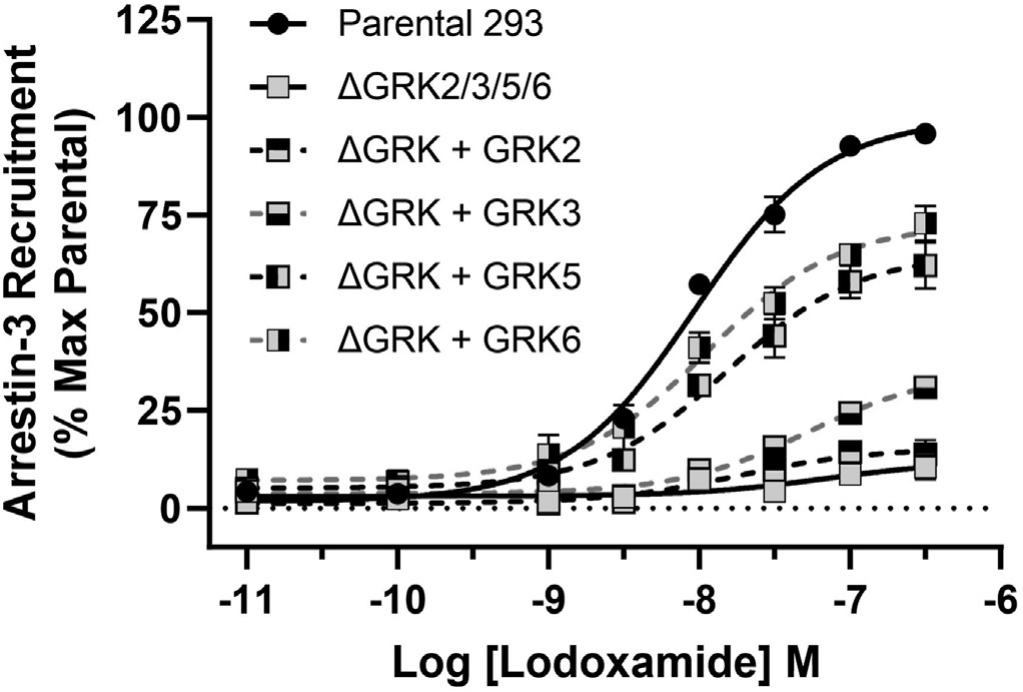
**Both GRK5 and GRK6 can allow agonist-mediated human GPR35a–arrestin-3 interactions.** ΔGRK2/3/5/6293 cells were transfected transiently with combinations of human GPR35a-eYFP and arrestin-3-RLuc and LgBiT-tagged forms of each of GRK2, GRK3, GRK5, and GRK6. Subsequently the ability of varying concentrations of lodoxamide to promote BRET signals reflecting GPR35a-eYFP-arrestin-3-RLuc proximity and/or interactions were recorded. Data for WT HEK293 and ΔGRK2/3/5/6 cells without the introduction of a GRK are provided for reference. Data are the mean ± SD of outcomes from three independent experiments. BRET, bioluminescence resonance energy transfer; GRK, G protein–coupled receptor kinase; HEK, Human embryonic kidney; LgBiT, large BiT; RLuc, *Renilla* luciferase.

**Figure 4:**
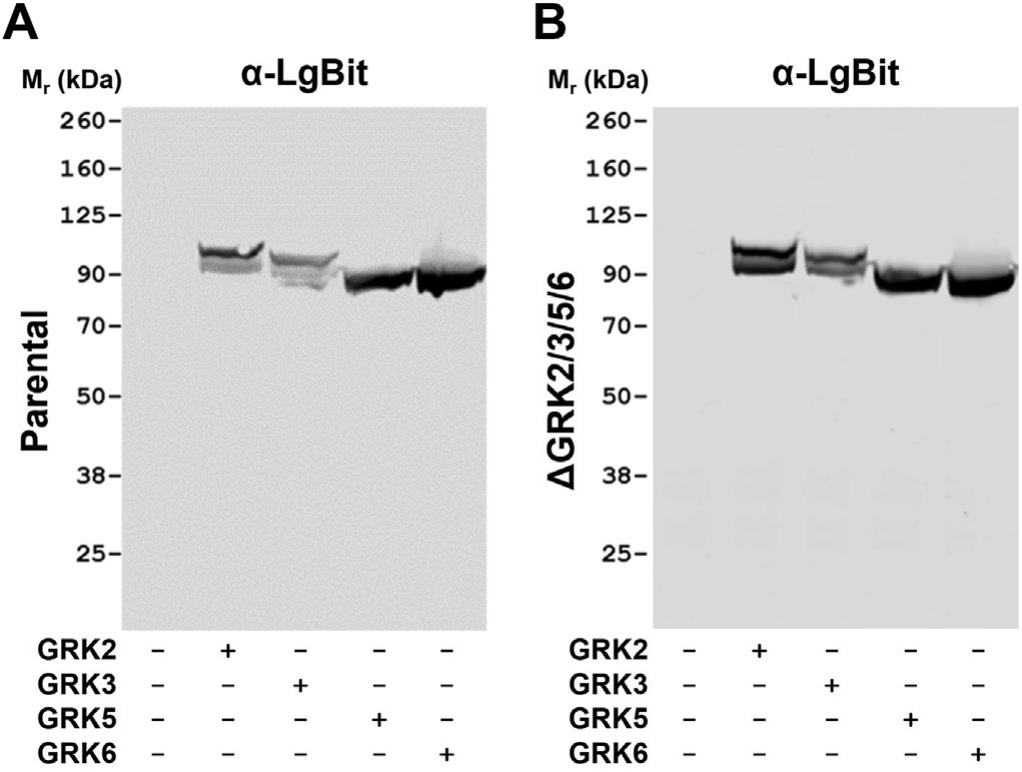
**Expression of introduced GRKs in HEK293-derived cell lines.** Following transfections akin to those in Figure 3, cell lysates were produced and resolved by SDS-PAGE. These (*A,* parental HEK293 and *B,* ΔGRK2/3/5/6 cells) were then immunoblotted with an anti-LgBiT antibody. The identity of the introduced GRK is noted in each case. GRK, G protein–coupled receptor kinase; HEK, Human embryonic kidney; LgBiT, large BiT.

In parental 293 cells lodoxamide-induced hGPR35a-eYFP/arrestin-3-RLuc interactions were inhibited by compound 19 in a concentration-dependent manner (pIC_50_ = 5.80 ± 0.06, mean ± SD, n = 3) (Fig. S4*A*) but unaffected by pretreatment with compound 101 (Fig. S4*B*). In ΔGRK2/3/5/6293 cells that had been transiently transfected with either GRK2 or GRK3 the limited extent of lodoxamide-induced hGPR35a-eYFP–arrestin-3-RLuc interactions was inhibited by pretreatment with compound 101 (pIC_50_ = 6.20 ± 0.59 [GRK2] and 6.13 ± 0.25 [GRK3], means ± SD, n = 3) (Fig. 5*A*). By contrast following introduction of GRK5 or GRK6 into ΔGRK2/3/5/6293 cells, lodoxamide-induced hGPR35a-eYFP/arrestin-3-RLuc interactions were again inhibited by compound 19 in a concentration-dependent manner with pIC_50_ = 5.34 ± 0.15 (GRK5) and 4.95 ± 0.29 (GRK6) (means ± SD, n = 3) (Fig. 5*B*).

**Figure 5:**
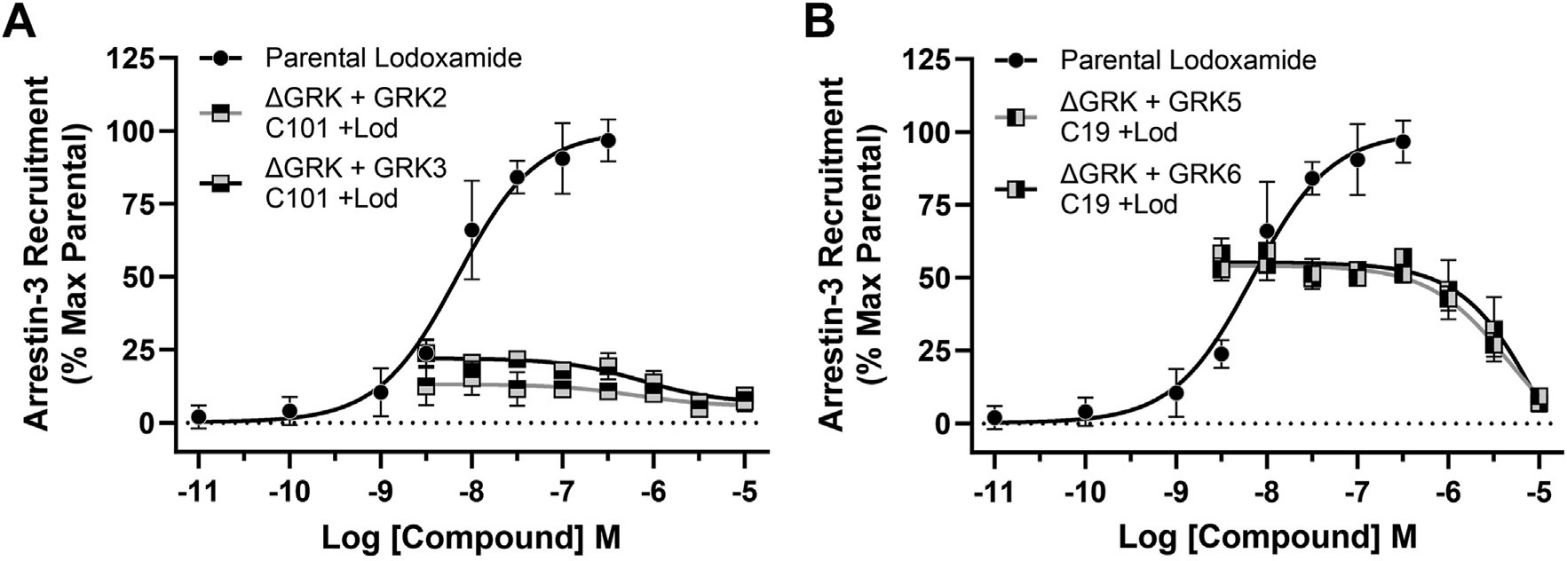
**Compound 19 prevents GRK5/6 mediated, lodoxamide-induced human GPR35a–arrestin-3 interactions in a concentration-dependent manner.** ΔGRK2/3/5/6293 cells were transfected coexpress hGPR35a-eYFP and arrestin-3-RLuc alongside GRK2 (*square, top filled*), GRK3 (*square bottom filled*) (*A*), or GRK5 (*square, left filled*) and GRK6 (*square, right filled*) (*B*). Prior to addition of lodoxamide (100 nM, 5 min) cells were pretreated for 30 min with varying concentrations of either compound 101 (GRK2 and GRK3 inhibitor) (*A*) or compound 19 (GRK5 and GRK6 inhibitor) (*B*). Data are presented as % of the effect of lodoxamide. In both *A* and *B*, a full concentration-response curve of lodoxamide-induced hGPR35a-eYFP–arrestin-3-RLuc interactions in parental 293 cells are shown for reference. Data are the mean ± SD of outcomes from three independent experiments. GRK, G protein–coupled receptor kinase; RLuc, *Renilla* luciferase.

### Inhibition of GRK5/6 but not GRK2/3 limits agonist-induced internalization of hGPR35a

Based on these outcomes, we next explored how these compounds might affect lodoxamide-induced internalization of hGPR35a from the surface of cells. To do so we established a cell line stably expressing hGPR35a with in-frame C-terminal attachment of Nano-luciferase (hGPR35a-Nluc). These cells were then transfected to express monomeric NeonGreen (mNG) that was plasma membrane targeted by N-terminal addition of the acylated 11 amino fragment of the N terminus of the nonreceptor tyrosine kinase Lyn (Lyn11-mNG). This provided a bystander BRET assay in which internalization of the receptor is anticipated to eliminate proximity of Nluc and mNG. Addition of lodoxamide produced concentration-dependent internalization of hGPR35a with pEC_50_ 8.26 ± 0.03 (Fig. 6*A*), while pretreatment with compound 19 limited (to 51.9 ± 4.5%, mean ± SD, n = 3), but did not ablate, this effect of lodoxamide (Fig. 6*B*). By contrast, pre-treatment with compound 101 was unable to limit lodoxamide-mediated receptor internalization (Fig. 6*B*). Pretreatment with both compound 101 and compound 19 was unable to cause greater inhibition of internalization than compound 19 alone (to 65.7 ± 8.3%, mean ± SD, n = 3) (Fig. 6*B*). While this assay is consistent with lodoxamide-induced receptor internalization it does not directly measure the movement of the receptor into a compartment within the cell. To address this specifically, we employed a second assay in which BRET is enhanced if the Nluc-tagged receptor moves to early endosomes as marked by a coexpressed mNG-FYVE construct. In this assay lodoxamide promoted BRET in a concentration-dependent fashion with pEC_50_ 8.03 ± 0.03 (Fig. 6*C*). Once more pretreatment with compound 19 (to 39.5 ± 5.0%, mean ± SD, n = 3), but not with compound 101 (112.2 ± 15.7%, mean ± SD, n = 3), limited but did not fully prevent receptor internalization and transfer to early endosomes (Fig. 6, *C* and *D*). Again, a combination of compound 19 and compound 101 was not more effective (to 36.2 2 ± 3.9%, mean ± SD, n = 3) in limiting hGPR35a internalization than compound 19 alone (Fig. 6*D*).

**Figure 6:**
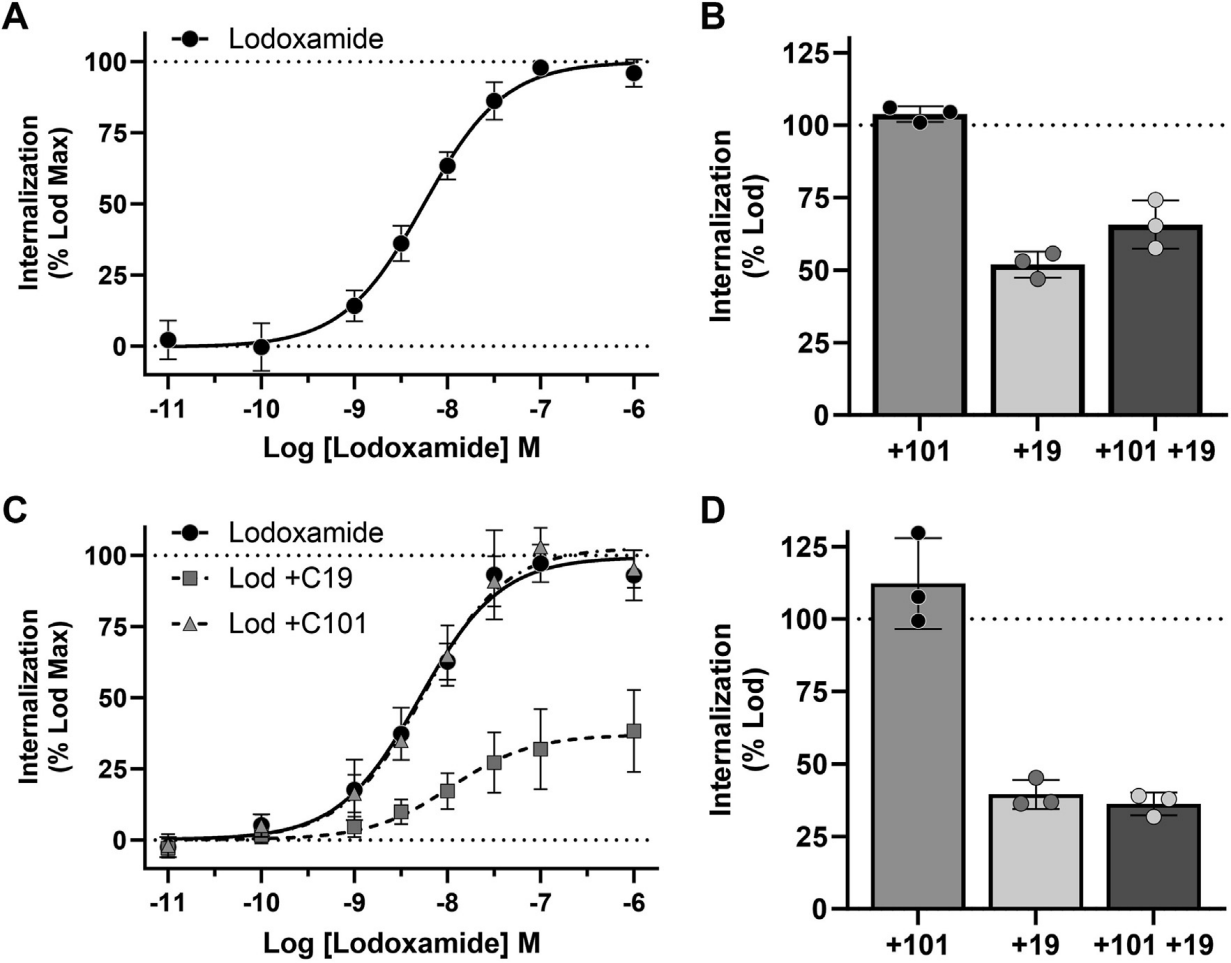
**GRK5 and/or GRK6 promote hGPR35a internalization.***A,* lodoxamide-induced internalization of hGPR35-Nluc expressed stably in Flp-In TREx 293 cells in a concentration-dependent manner. Studies employed the loss of proximity of hGPR35-Nluc and Lyn11-mNG as a measure of movement of hGPR35-Nluc away from the plasma membrane. *B,* lodoxamide (100 nM)-induced internalization of hGPR35-Nluc was reduced by pretreatment with compound 19 but not by compound 101 (each at 10 μM). Pretreatment with a combination of compound 19 and compound 101 did not further reduce lodoxamide-induced internalization of GPR35. *C,* lodoxamide induced movement of hGPR35-Nluc to early endosomes as assessed by the increased proximity of hGPR35-Nluc to mNG-FYVE. *D,* this effect of lodoxamide (100 nM) was also suppressed by pretreatment of cells with compound 19 but not compound 101. Error bars represents SD of means of three separate experiments. GRK, G protein–coupled receptor kinase; mNG, monomeric NeonGreen; Nluc, nano-luciferase.

### Inhibition of GRK5/GRK6 prevents agonist-mediated phosphorylation of hGPR35a at amino acids Ser^300^ and Ser^303^

We have previously characterized each of the hydroxyamino acids in the intracellular C-terminal tail of hGPR35 that contribute to interactions with arrestins (5). Although mutation of each of Ser^287^, Ser^300^, Ser^303^, and Thr^307^ individually to Ala was shown to have significant impact, combination of mutation of Ser^300^ and Ser^303^ produced a form of the receptor with little capacity to interact with arrestin-3 (5). As part of these earlier studies, we generated an antiserum that identifies specifically pSer^300^-pSer^303^ hGPR35a and showed that this antiserum only identifies hGPR35a postagonist activation (5).

We next generated a stably transfected Flp-In TREx 293 cell line that is induced to express a C terminally HA (haemagglutinin)-tagged form of hGPR35a (hGPR35a-HA) by exposure to doxycycline. Addition of lodoxamide (100 nM, 5 min), followed by capture of the receptor using HA-trap, allowed for detection of the receptor in immunoblots by the anti-pSer^300^-pSer^303^ antiserum (Fig. 7*A*). As previously reported (5), this was as a pair of well-resolved species that reflect differential N-glycosylation (Fig. 7*A*). Pretreatment of the cells with compound 19 fully prevented lodoxamide-induced pSer^300^-pSer^303^ phosphorylation (Fig. 7*A*). By contrast, treatment with compound 101 reduced, but did not ablate, recognition by the anti-pSer^300^-pSer^303^ antiserum (Fig. 7*A*). Now, in addition, we generated an antiserum against the distal part of the C-terminal tail of hGPR35a. Although designed as a structural antiserum for which recognition of the receptor protein was anticipated to be independent of receptor activation/phosphorylation state, this was not the observed characteristic of this reagent (Fig. 7*B*). In parallel immunoblots, treatment with lodoxamide reduced recognition of hGPR35a-HA by this antiserum (Fig. 7*B*). This may indicate that lodoxamide-induced incorporation of negative charge, reflecting phosphorylation of residues including Ser^300^ and Ser^303^, may interfere with epitope recognition by the antiserum. In support of this concept, pretreatment of cells with compound 19 prior to addition of lodoxamide did not result in such a decline in recognition of hGPR35a-HA by this antiserum (Fig. 7*B*). In comparison pretreatment with compound 101 prior to addition of lodoxamide produced a limited reduction in the effect of lodoxamide (Fig. 7*B*). To confirm these observations were not loading artefacts, or that the treatment with lodoxamide in the presence or absence of the GRK inhibitors had not altered the total amount of hGPR35a-HA protein, we performed additional parallel immunoblots using an anti-HA antibody. These showed equivalent levels of the receptor construct across experimental conditions (Fig. 7*C*).

**Figure 7:**
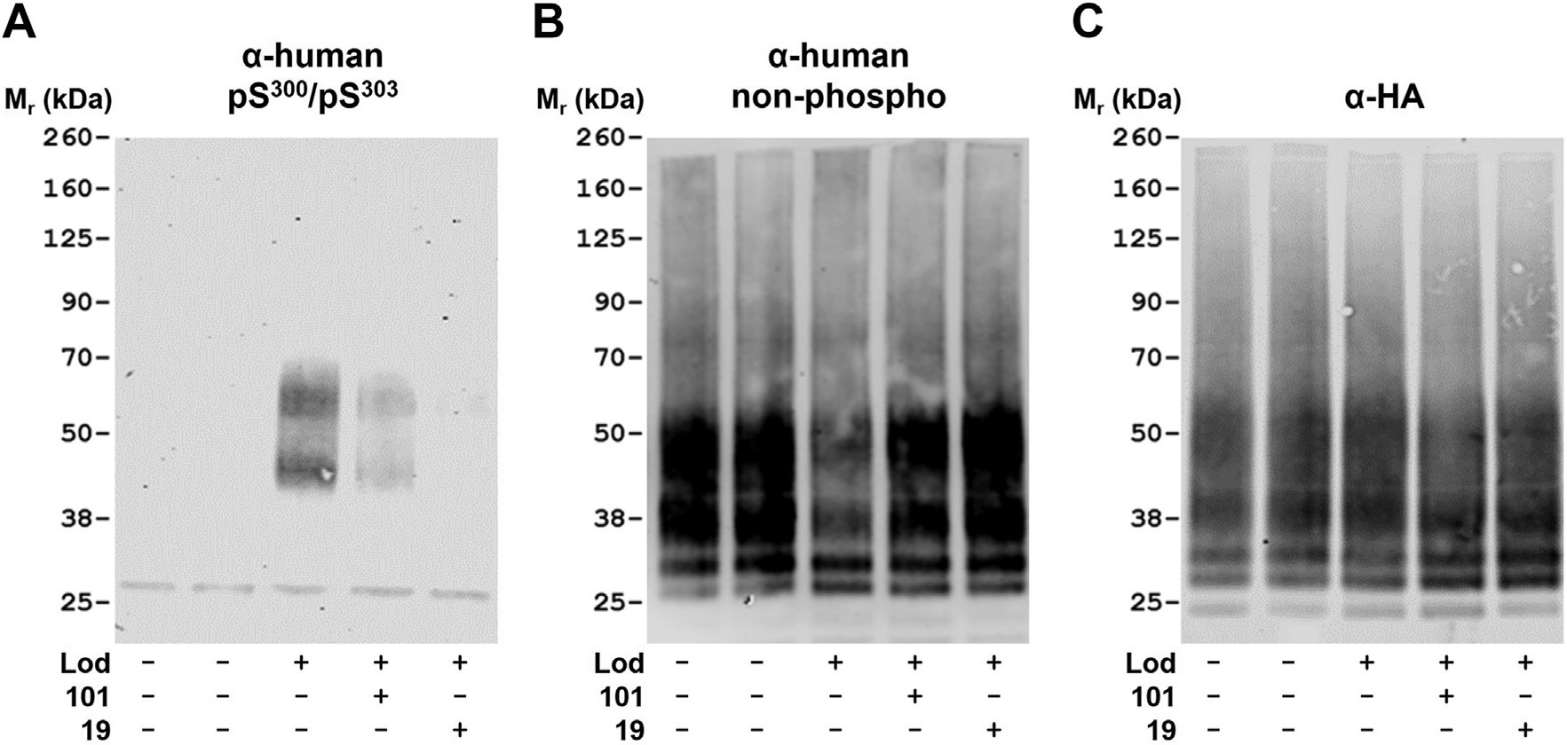
**Phosphorylation of human GPR35a at pSer**^**300**^**-pSer**^**303**^**is prevented by compound 19.** A Flp-In TREx 293 cell line induced to express human GPR35a-HA (*all lanes*) was treated with lodoxamide (100 nM) (Lod+) or vehicle ((Lod−), lodoxamide plus compound 101 (101+) or lodoxamide plus compound 19 (19+) (each inhibitor at 10 μM, added 30 min before lodoxamide). Subsequently, after anti-HA immunoprecipitation, samples were resolved by SDS-PAGE and immunoblotted to detect (*A*) human GPR35a-pSer^300^-pSer^303^, (*B*) the C-terminal region of GPR35a, or (*C)* the HA epitope tag. Results are representative of three independent experiments. HA, haemagglutinin.

We wished to explore this aspect more fully. There are additional potential sites of phosphorylation in the C-terminal tail of mouse GPR35 compared to human (5). However, amino acids equivalent to Ser^300^ and Ser^303^ (Ser^298^ and Ser^301^ in the mouse orthologue) are conserved and their phosphorylation is equally, if not even more, important for interactions with arrestin-3 (5). We hence performed a limited set of studies using mouse GPR35-HA. Here we used a distinct phospho-specific antiserum that specifically identifies mouse GPR35 pSer^298^-pSer^301^ (5). In SDS-PAGE performed on lysates of stably transfected Flp-In TREx 293 cells induced to express mouse GPR35-HA, as reported previously (5), the receptor protein migrated as an apparently single species of some 50 kDa and phosphorylation of pSer^298^-pSer^301^ was entirely dependent upon treatment with the mouse GPR35 active agonist zaprinast (Fig. 8*A*). We selected zaprinast for these studies both because this was the ligand used by (5) for earlier studies on the mouse orthologue and because lodoxamide has only modest potency at mouse GPR35 (12). Pretreatment with compound 19 prior to addition of zaprinast fully eliminated agonist-induced recognition of mouse GPR35-HA by the anti-pSer^298^-pSer^301^ antiserum (Fig. 8*A*). Even more clearly than when using hGPR35a-HA, the nonphospho-site GPR35 C-terminal tail antiserum almost entirely failed to identify mGPR35-HA after treatment with zaprinast (Fig. 8*B*). Pretreatment with compound 19 fully prevented the zaprinast-induced loss of recognition by this antiserum (Fig. 8*B*). Unlike the partial effect in cells expressing hGPR35-HA, here compound 101, although again partially limiting detection of mouse GPR35-HA by the mouse GPR35 pSer^298^-pSer^301^ antiserum (Fig. 8*A*), did not partially restore identification of mouse GPR35-HA by the structural anti-GPR35 antiserum (Fig. 8*B*). Once more, parallel immunoblots with anti-HA confirmed equivalent loading of samples onto the gels (Fig. 8*C*).

**Figure 8:**
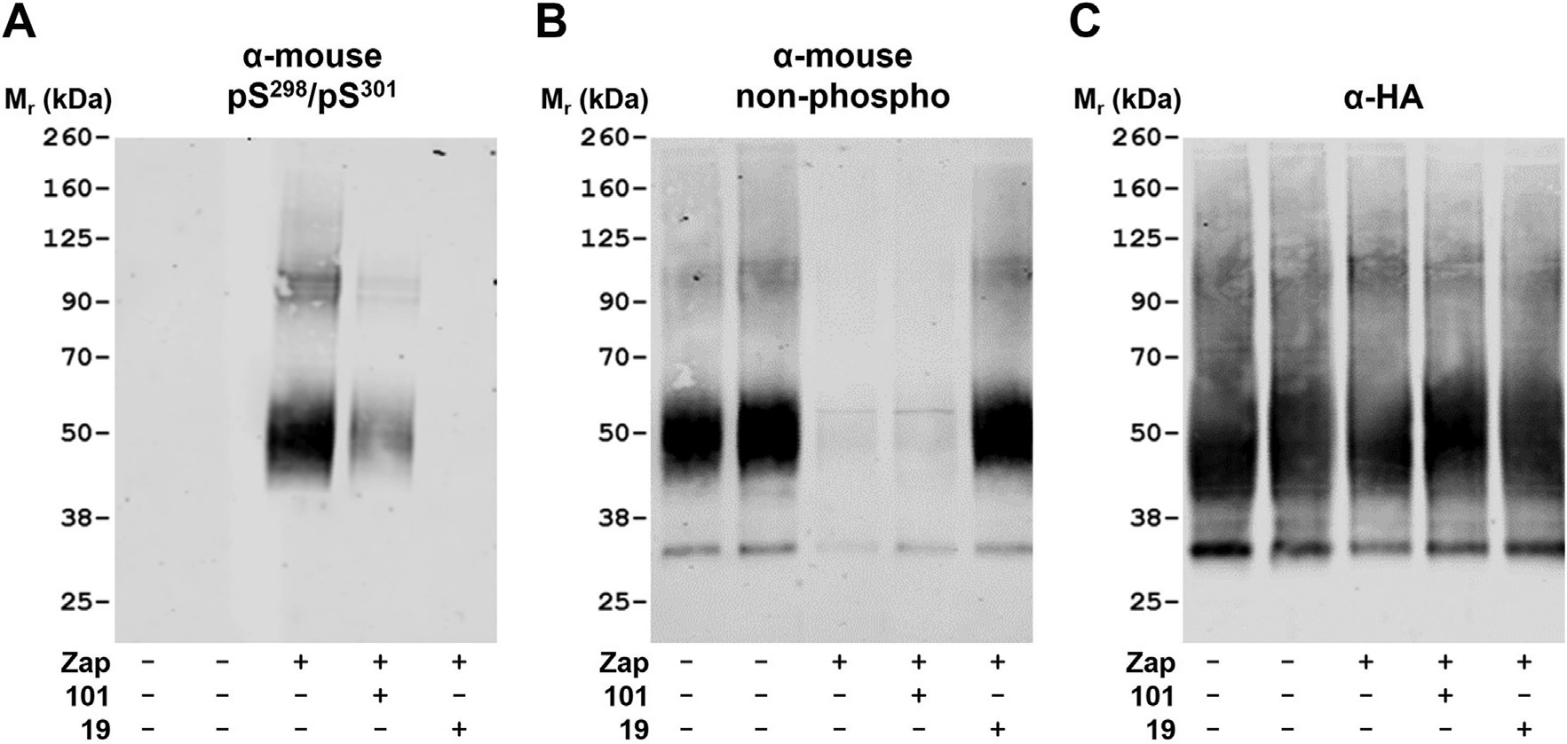
**Recognition of agonist-activated mouse GPR35 by an anti-mouse GPR35-pSer**^**298**^**-pSer**^**301**^**antiserum is prevented by inhibition of GRK5/6.** Experiments akin to Figure 7 were performed using a Flp-In TREx 293 cell line induced to express mouse GPR35-HA (all lanes). Zaprinast (10 μM) (Zap+), which is an agonist at mouse GPR35, replaced lodoxamide. In some samples zaprinast was replaced by vehicle (Zap−). Pretreatment with compound 101 (101+) or compound 19 (19+) (each at 10 μM) was for 30 min. Immunoblots of anti-HA immunoprecipitated samples are shown. (*A*) mouse GPR35a-pSer^298^-pSer^301^, (*B*) the C-terminal region of GPR35a, or (*C*) the HA epitope tag. Results are representative of three independent experiments. GRK, G protein–coupled receptor kinase; HA, haemagglutinin.

The human pSer^300^-pSer^303^ GPR35a and mouse pSer^298^-pSer^301^ GPR35 antisera also detect the corresponding receptors in an agonist-dependent manner in immunocytochemical studies (5). In Flp-In TREx 293 cells induced to express either hGPR35a-HA or mouse GPR35-HA pretreatment with compound 19 prevented immunocytochemical detection of the receptors by these antisera that was induced by addition of appropriate agonist ligands (Fig. 9). Parallel detection of each orthologue by anti-HA confirmed that the loss of agonist-induced detection by the phospho-site–specific antisera in the presence of compound 19 did not reflect loss of receptor protein (Fig. 9).

**Figure 9:**
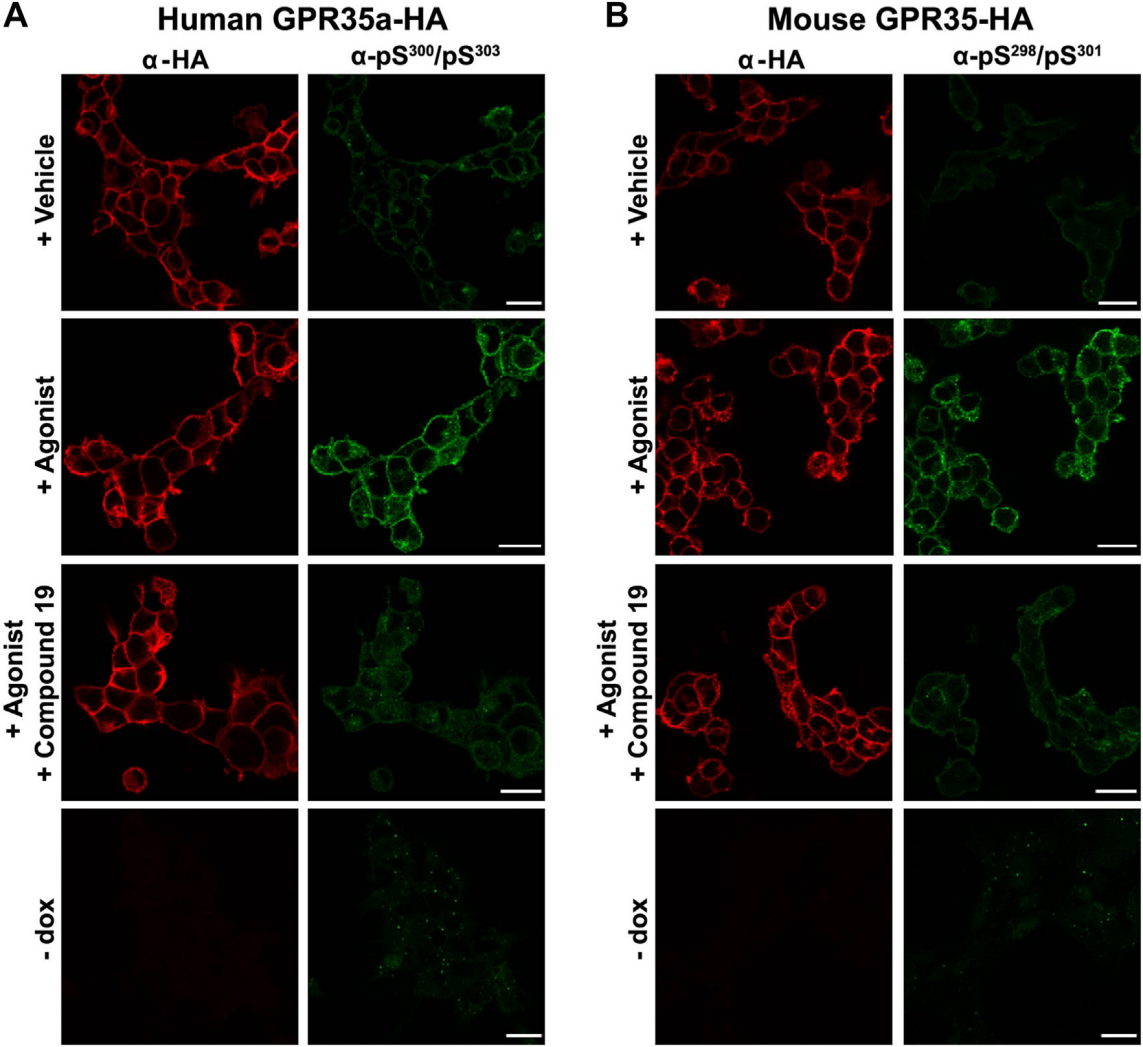
**Agonist-induced immunocytochemical detection of both human and mouse GPR35 is prevented by inhibition of GRK5/6.** Flp-In TREx 293 cell lines as in Figures 7 and 8 were induced to express either human GPR35a-HA (*A*) or mouse GPR35-HA (*B*) or, as a control were uninduced (−dox). The induced cells were subsequently treated with vehicle, or an appropriate GPR35 agonist (human, lodoxamide 100 nM, mouse, zaprinast 10 μM) for 5 min, without or with pretreatment with compound 19 (10 μM) for 15 min. After fixation cells were immunostained with anti-HA (*red*) or human (pSer^300^-pSer^303^) GPR35a (*A*) or mouse (pSer^298^-pSer^301^) GPR35 (*B*) (*green*). Preaddition of compound 19 prevented identification of each species ortholog of GPR35 by the corresponding phospho-site–specific antiserum. The scale bar represents 20 μm. GRK, G protein–coupled receptor kinase; HA, haemagglutinin.

### GRK5 and GRK6 directly produce agonist-induced phosphorylation of hGPR35a Ser^300^-Ser^303^

We now returned specifically to studies on hGPR35a. We transiently introduced LgBiT-tagged forms of GRK2, GRK3, GRK5, or GRK6, along with hGPR35-HA, into ΔGRK2/3/5/6293 cells. Cells were treated with lodoxamide (100 nM, 5 min), and the receptor was then captured using anti-HA-beads. Subsequent immunoblots with anti-HA confirmed equivalent transfection and capture of the receptor (Fig. 10*A*). Parallel immunoblots with anti-hGPR35 pSer^300^-pSer^303^ indicated that phosphorylation of these sites was promoted by the presence of GRK5 and, particularly, GRK6 but that GRK2 and GRK3 were without measurable effect (Fig. 10*B*). Such effects of GRK5 and GRK6 were fully attenuated if cells had been pretreated with compound 19 (Fig. 10*C*).

**Figure 10:**
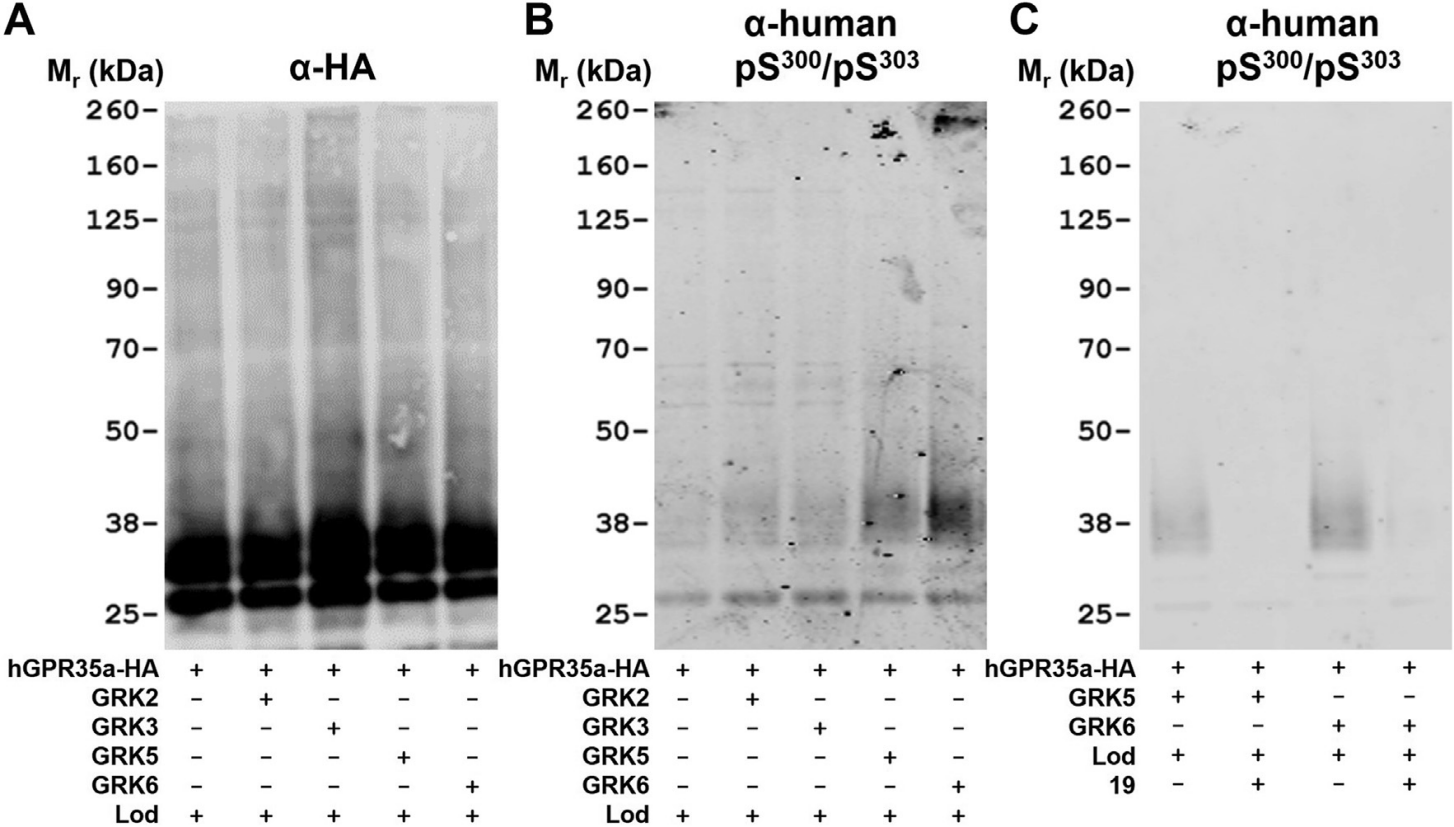
**GRK5 and GRK6 directly promote hGPR35 phosphorylation at Ser**^**300**^**and Ser**^**303**^**.** ΔGRK2/3/5/6 cells were transfected transiently with combinations of human GPR35a-HA (*all lanes*) and LgBiT-tagged forms of individual GRK isoforms as noted (+). After treatment with lodoxamide (100 nM, 5 min) (Lod+) samples were captured with anti-HA beads and resolved by SDS-PAGE. These were subsequently immunoblotted to detect (*A*) the HA epitope tag or (*B*) pSer^300^-pSer^303^-hGPR35a-HA. In similar studies (*C*), ΔGRK2/3/5/6 cells coexpressing hGPR35a-HA and LgBiT-tagged forms of either GRK5 or GRK6 (+) were treated without (−) or with (+) compound 19 (10 μM, 30 min) ahead of exposure to lodoxamide. Anti-HA bead immunoprecipitated samples were immunoblotted with the pSer^300^-pSer^303^-hGPR35a-HA antiserum after SDS-PAGE. *Note*: the lower apparent molecular mass of hGPR35a-HA following transient transfection compared to when expressed stably (Figs. 7 and 8). This may reflect differing degrees of maturation and posttranslational modification. GRK, G protein–coupled receptor kinase; HA, haemagglutinin; LgBiT, large BiT.

Finally, we attempted to assess whether activation of GPR35 altered the interactions or proximity of the receptor with either GRK5 or GRK6. To do so, we transiently introduced the LgBit-tagged form of GRK5 and GRK6 (20) into HEK293 cells alongside a C terminally small Bit (SmBit)–tagged form of GPR35. As anticipated from the known plasma membrane association of GRK5 and GRK6, this resulted in marked luciferase activity in the basal state following addition of substrate (Fig. 11*A*). Notably, addition of lodoxamide resulted in a marked, concentration-dependent, reduction in luciferase activity for both GRK5 and GRK6. The observed potency of lodoxamide (range pEC_50_ 7.58–7.90 in various assays) was similar to that observed for arrestin-2 and arrestin-3 recruitment to hGPR35a. Notably, pretreatment with compound 19 blunted, but did not eliminate, the effect of lodoxamide in this assay (Fig. 11*B*).

**Figure 11:**
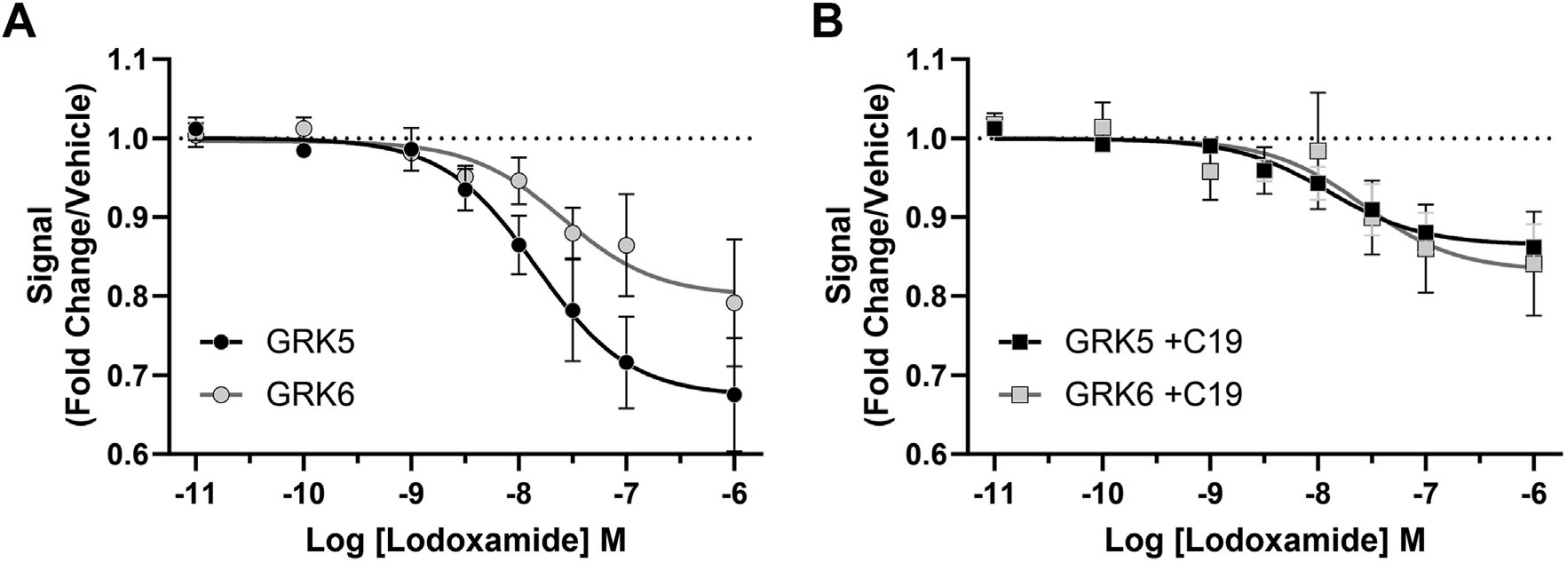
**Lodoxamide alters the proximity of hGPR35a with coexpressed GRK5 and GRK6.** ΔGRK2/3/5/6 cells were transfected transiently with hGPR35a-SmBit and LgBiT-tagged forms of GRK5 or GRK6 isoforms as noted and subsequently exposed to various concentrations of lodoxamide for 5 min. In *B* but not *A* cells were pretreated with compound 19 (10 μM) for 30 min before exposure to lodoxamide. Nluc complementation as a measure of the proximity of hGPR35a and each GRK was then measured. Error bars represents SD of means of three separate experiments. GRK, G protein–coupled receptor kinase; LgBiT, large BiT; Nluc, nano-luciferase.

## Discussion

The importance of members of the GRK family in GPCR regulation and in promoting interactions with arrestin adaptor proteins has been known for many years (2, 17, 24). Despite this, information on the identity of GRKs that interact with and regulate individual receptors and particular phosphorylated residues is sparse. Furthermore, how phosphorylation of specific serine and/or threonine residues within the intracellular elements of GPCRs relate to the concept of phosphorylation “bar-coding” is still unclear. This, however, may underlie how subsequent cellular responses to agonists can vary in different cell types and tissues (7, 10). There have, however, been recent advances that are contributing to addressing these issues. Key among these has been the ability to define sites of regulated phosphorylation by unbiased mass spectrometry (25, 26). The production of genome-edited cell lines lacking expression of one or more of the ubiquitously expressed GRK isoforms, GRK2, GRK3, GRK5, and GRK6 (13), also offers new approaches to functional reconstitution and definition of GRK selectivity. In addition, the development of both selective small molecule GRK inhibitors (14, 18) and similarly, the development of phospho-site–specific antisera that detect sites of regulated or constitutive phosphorylation within GPCRs (5, 6, 9, 27, 28) have been providing new insights.

Herein, we built on previous work in which we defined the sites of agonist-regulated phosphorylation in GPR35 (5). This receptor can be activated by the endogenously produced tryptophan metabolite kynurenic acid and by a considerable number of synthetic ligands (12) but is still officially designated as an orphan GPCR (11). Despite this, significant expression in crypts of the colon and a clear association between a single nucleotide polymorphism that results in a T108M variation in transmembrane III of the protein and inflammatory diseases of the lower gut (11) has promoted substantial interest in the concept that agonists of GPR35 might be effective in the treatment of ulcerative colitis and related disorders (12). As such, a clear understanding of the regulation of GPR35 by GRK isoforms was, herein, a key objective.

In earlier studies, we showed the importance of Ser^300^ and Ser^303^ in human GPR35a for agonist-mediated interactions with arrestin isoforms (5) and within these studies developed and used a pSer^300^/pSer^303^ antiserum, which identified this receptor in an entirely agonist/activation-dependent fashion. We have also used this phospho-site–specific antiserum as well as a second one that targets the equivalent phosphorylated residues in mouse GPR35 (5) in the current studies. In addition, herein, we also employed an antiserum raised against the nonposttranslationally modified C-terminal tail of GPR35 which, particularly for mouse GPR35, lost recognition of the receptor upon agonist treatment. By introducing in-frame a C-terminal HA epitope tag sequence into both human GPR35a and mouse GPR35, we were however able to identify the receptors in a manner unaffected by agonist treatment.

Although compound 101 is a well-studied and well-established selective inhibitor of GRK2 and GRK3 (18, 19), until recently there has been limited options for selective small molecule inhibition of GRK5 and GRK6. The set of quinazoline-based inhibitors described by Uehling *et al*. (14) has, however, offered a means to address this. Compound 19, and others from this series, effectively blocked detection of agonist-mediated phosphorylation of both the human and mouse orthologue by the (human GPR35) pSer^300^/pSer^303^ antiserum and the equivalent mouse receptor-directed antiserum in both immunoblotting and immunocytochemistry studies, as well as preventing agonist-induced interactions of human GPR35a with either arrestin-2 or arrestin-3. In addition, the availability of 293-derived cell lines lacking expression of various GRKs (13) demonstrated that lack of both GRK2 and GRK3 did not inhibit agonist-induced receptor–arrestin-3 interactions, while lack of both GRK5 and GRK6 almost fully prevented such interactions. Moreover, the ΔGRK2/3/5/6 HEK293 cells allowed reconstitution studies with individual GRK isoforms and these demonstrated that GRK5 and GRK6 were essentially equi-effective. We were also able to demonstrate that inhibition of GRK5/6, but not GRK2/3, limited agonist-induced internalization of hGPR35a in two distinct assays, again consistent with the selective effect of the small molecule inhibitors on agonist-induced interactions with the arrestin isoforms. However, inhibition of GRK5/6 did not eliminate hGPR35a internalization/movement to early endosomes, suggesting that there may be a GRK-independent component for this receptor, at least in HEK293 cells. This is perhaps surprising as we failed to observe internalization of this receptor in response to a different GPR35 agonist in cells genome-edited to lack expression of arrestin isoforms (5). Our exploration of how proximity and functional reconstitution of coexpressed SmBit-tagged hGPR35a and LgBit-tagged forms of GRK5 and GRK6 was affected by lodoxamide-induced activation of the receptor resulted in an additional surprising outcome. A clear reduction in luciferase signal was observed for each GRK and the concentration dependence for lodoxamine was very similar to that observed to promote hGPR35a and arrestin-2/arrestin-3 proximity. The implications of these results are not entirely clear, but it should be noted that similar reductions in signal when using these GRK5 and GRK6 constructs have been reported for other GPCRs, including the μ-opioid receptor, the β_2_-adrenoceptor, and the atypical, arrestin-biased chemokine receptor ACKR3 (20).

In total, the cell-based studies illustrated key roles for GRK5 and GRK6, with lesser if any contribution of GRK2 and/or GRK3, to phosphorylation of these specific sites or to interaction of GPR35a with arrestins. To attempt to consider this at a more mechanistic and molecular level, we examined the recently reported cryo-EM structure of GPR35a in association with a chimeric G protein containing the receptor-interacting α5 helix of Gα_13_ and the agonist ligand lodoxamide (29). Although there are some potential issues with the reported position of the agonist ligand, and the possible role of a co-ordinated cation in the ligand binding pocket in this structure, on the intracellular face of the receptor the structure highlights an unusual “methionine pocket” that accommodates the Gα_13_ C-terminal helix (29). Examination of predictions of this using the “AlphaFold” deep learning algorithm (30) provides a highly similar view of the organization of this region (Fig. 12*A*). Similarly, this approach suggests that the extreme N-terminal region of GRK5 occupies this same methionine pocket (Fig. 12*B*). However, although the N terminus of GRK6 overlays with GRK5 in such models, neither GRK2 nor GRK3 do so (Fig. 12*C*). Such models are at a minimum consistent with the GRK-selectivity profile produced from the experimental studies.

**Figure 12:**
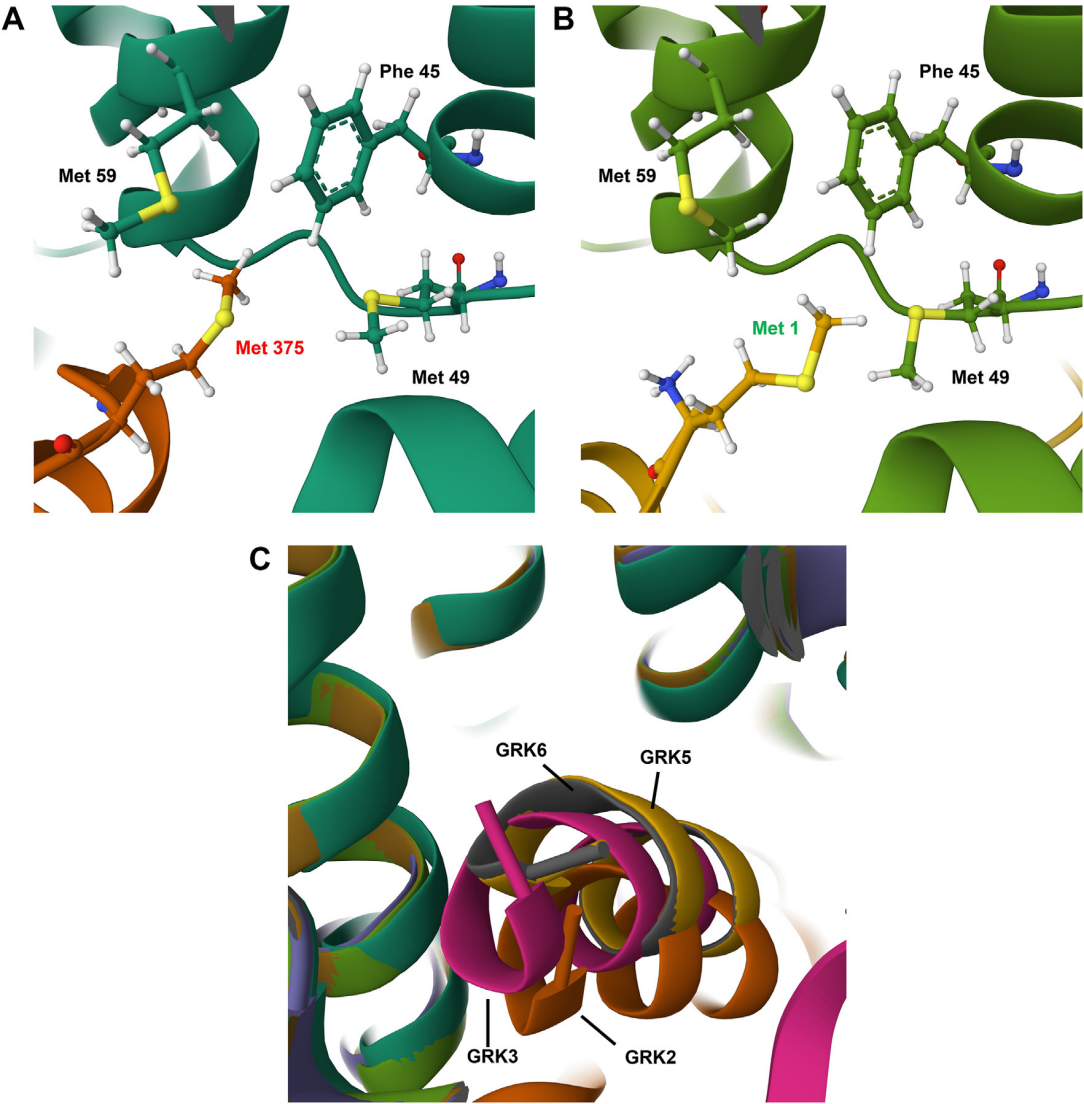
**AlphaFold models of GPR35-GRK selectivity suggest selective interactions with GRK5/6.** AlphaFold predictions of the positioning of (*A*) the C-terminal portion of the α5 helix of Gα_13_ (*orange*) within the methionine pocket on the intracellular face of GPR35a (*green*). Met 375 of Gα_13_ is located three amino acids from the C terminus. *B,* Met 1 of GRK5 is predicted to occupy equivalent space. Similar outcomes were observed for GRK6. *C,* AlphaFold modeling predicts distinct peptide positioning and alignment of GRK2 (*orange*) and GRK3 (*pink*) isoforms compared to GRK5 (*yellow*) or GRK6 (*gray*), consistent with agonist-induced GPR35 selectivity. GRK, G protein–coupled receptor kinase.

As shown in the initial stages of these studies a phospho-deficient form of hGPR35a in which all five potential phospho-acceptor sites in the C-terminal tail are altered to Ala failed to interact with arrestin-3 in an agonist-dependent manner. Modeling of this region of the receptor shows that in an inactive state the PDM C terminal is likely to be located in a similar position to the equivalent region of the WT receptor (Fig. 13*A*). However, when we modified this sequence *in silico* to alter the potential phospho-acceptor amino acids to the phospho-mimetic Asp, this variant now interacted directly with arrestin-3 (Fig. 13*B*) in a groove shown in structural studies to allow interaction between the tail of the vasopressin V2 receptor and arrestin-2 (31). Based on such studies, broader understandings of the likely basis of GPCR–arrestin interactions were developed (32), even ahead of direct observations (33)

**Figure 13:**
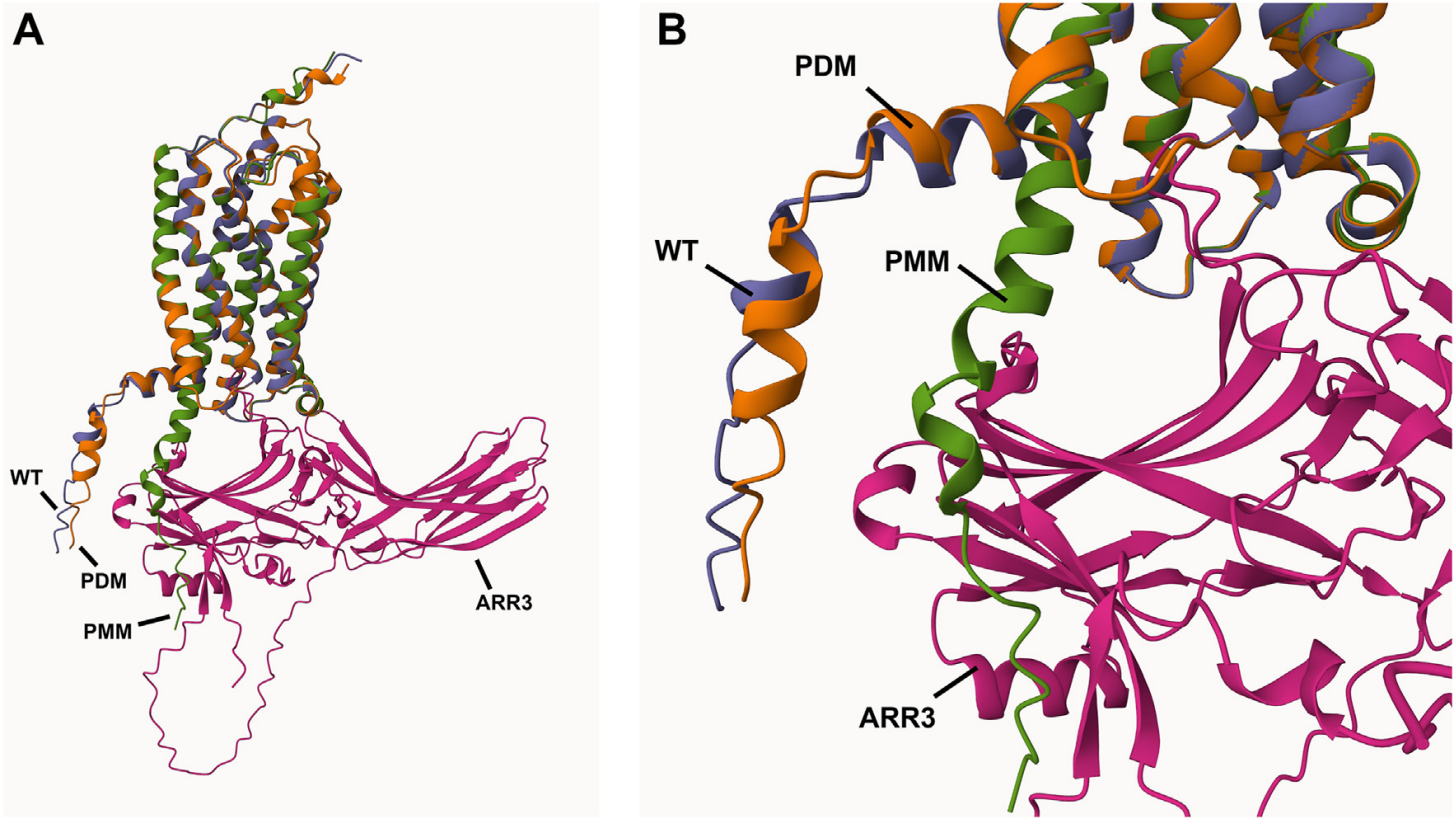
**AlphaFold model of the interaction of a phospho-site mimetic peptide of the C-terminal tail of GPR35 shows direct interactions with arrestin-3.** AlphaFold models of human GPR35 in association with arrestin-3 (ARR3). Variations in the C-terminal tail from the WT (*blue*) sequence were the PDM variant used in the experimental studies in which all five phospho-acceptor amino acids were converted to Ala (*orange*) and a phospho-site mimetic (PMM) (*green*) in which these residues were each altered to Asp. *A,* full model of GPR35 and arrestin-3. *B,* focus on regions of interaction. PDM, phospho-acceptor site–deficient mutant.

One element that requires comment is that for both human GPR35a and mouse GPR35, while pretreatment with compound 19 fully prevented recognition of the receptor after agonist stimulation by the phospho-site–specific antisera used herein, pretreatment with the GRK2/GRK3 inhibitor compound 101 partially limited such recognition. This might be thought inconsistent with the lack of effect of compound 101 on arrestin recruitment. While we do not have a definitive explanation for this, as noted earlier there are five phospho-acceptor sites in the C-terminal tail of human GPR35a (and indeed nine in the C-terminal tail of mouse GPR35). We have shown previously that although phosphorylation of Ser^303^ is of particular importance for interactions with arrestins (5) all five residues in the human receptor can be phosphorylated in an agonist-dependent manner. Moreover, we observed that the antisera developed to identify the nonmodified form of the receptor C-terminal tail, identified human GPR35a less well, and mouse GPR35 very poorly indeed, after agonist treatment and subsequent phosphorylation. It is thus possible that GRK2/3 may phosphorylate one or more of the other phospho-acceptor residue(s) and that this may impair epitope recognition by the pSer^300^/pSer^303^ antiserum. Indeed, the antigen used to generate the human antiserum (KAHKpSQDpSLCVTL) contains a further phospho-acceptor residue, while the mouse GPR35–directed pSer^298^/pSer^301^-GPR35 antiserum was raised against the sequence TPHKpSQDpSQILSLT, which contains an additional two phospho-acceptor residues.

The current studies illustrate the importance of GRK5 and GRK6 in promoting agonist-dependent phosphorylation of, at a minimum, residues Ser^300^ and Ser^303^ in human GPR35a (and residues Ser^298^ and Ser^301^ in the mouse orthologue). Based on mutational analysis these residues are central to agonist-induced interaction of these orthologues of GPR35 with arrestins (5). However, it is important to reflect that all the reported studies have used WT and genome-engineered HEK293–derived cells. There is growing understanding that distinct kinases may promote differential phosphorylation patterns in different cell types and tissues (1) and it is now appreciated that each of the ubiquitously expressed arrestin isoforms can interact in a distinct manner with the same GPCR based on phosphorylation pattern (34). This is likely to generate a level of functional selectivity that may help to account for why the same ligand-receptor pairing can produce different outcome in different cells and tissues. Effort now needs to be directed to define if such differential patterning is actually produced in physiologically relevant tissues and, if so, how this may impact on cell type and tissue function.

## Experimental procedures

### Experimental

#### Materials

Lodoxamide, zaprinast, and compound 101 were purchased from Tocris Bioscience. Compounds 15, 16, 17, and 19 were described in Uehling *et al*. (14). Cell culture reagents were purchased from Thermo Fisher Scientific. PEI (linear poly (vinyl alcohol) [MW-25000]) was from Polysciences. cOmplete EDTA-free Protease Inhibitor Cocktail and PhosSTOP Phosphatase Inhibitor Cocktail were purchased from Roche Diagnostics.

### Antibodies/antisera

A rabbit phospho-site–specific hGPR35 antiserum pSer^300^/pSer^303^-hGPR35a (catalogue number 7TM0102C), raised against the sequence KAHKpSQDpSLCVTL, and a pSer^298^/pSer^301^-mGPR35 antiserum (7TM0102B), raised against the sequence TPHKpSQDpSQILSLT have been described previously (5). A GPR35 (nonphospho) antibody (cat number 7TM0102N), directed against the distal part of the carboxyl-terminal tail of hGPR35 was developed in collaboration with 7TM Antibodies GmbH. IRDye 800CW donkey anti-rabbit immunoglobulin G (IgG), IRDye 800CW donkey anti-goat IgG, and IRDye 800CW goat anti-rat IgG were from LI-COR Biosciences. Horseradish peroxidase anti-mouse (sheep) was from GE Healthcare. High-affinity anti-HA (rat) and anti-HA affinity matrix were from Roche Diagnostics. Anti-LgBiT mAb (cat number N7100) which is an affinity-purified mouse mAb for detection of LgBiT and LgBiT-fusion proteins by Western blotting was purchased from Promega Corporation. GRK isoform–directed antisera for GRK 2 sc-13143 (C-9) c and GRK 5 sc-518005 (D-9) were from Santa Cruz Biotechnology while GRK 3 CS #80362 (D8G6V) and GRK 6 CS #5878 (D1A4) were from Cell Signaling Technology. Further details are found in Reichel *et al*. (23).

### Generation of constructs

Generation of FLAG-hGPR35a-eYFP, hGPR35a-HA, FLAG-mGPR35-eYFP, and mGPR35-HA have been described previously (35, 36). The Stratagene Quik Change method (Stratagene, Agilent Technologies) was used to introduce alterations into each of the above constructs to produce both point mutants and phospho-deficient variants. Primers utilised for mutagenesis were from MWG Operon. Sequencing was carried out to confirm the introduction of the alterations.

### Construction of arrestin-3–Rluc

A full-length cDNA encoding Rluc 6 (312 amino acids) (37), generated by PCR amplification of a Rluc-containing vector plasmid (pRLCMV), was digested with XbaI and NhoI, and the resulting 1-kilobase XbaI-NhoI fragment was subcloned into a bovine arrestin-3-pcDNA3 vector, resulting in arrestin-3-RLuc-pcDNA3.

### Maintenance of cell lines

HEK 293T cells, GRK parental and genetically engineered (ΔGRK2/3/5/6), (ΔGRK2/3), (ΔGRK5/6) lines (13) were maintained in Dulbecco’s modified Eagle’s medium (DMEM) supplemented with 0.292 g/l L-glutamine, 1% penicillin/streptomycin mixture, and 10% heat-inactivated fetal bovine serum (FBS) at 37 °C in a 5% CO_2_ humidified atmosphere. Flp-In TREx 293 cells (Thermo Fisher Scientific) were maintained in DMEM without sodium pyruvate, supplemented with 10% FBS, 1% penicillin/streptomycin mixture, and 10 μg/ml blasticidin at 37 °C in a 5% CO_2_ humidified atmosphere.

### Transient transfection

PEI-mediated transient transfection was used as the default method. For a 10 cm^2^ culture dish, 5 μg of DNA was diluted in 250 μl 150 mM NaCl and mixed 1:1 with 250 μl 150 mM NaCl containing 30 μg PEI. The mixture was vortexed for 10 s and incubated for 10 min at room temperature before adding dropwise to the dish. Cells were incubated with the PEI overnight at 37 °C, then transfection medium was replaced with fresh culture medium. Cells were incubated for a further 24 to 48 h before use in assays. Transfection of HEK293 and the lines derived from them using this method was routinely above 80%. We did not observe substantial differences between the various lines used in the study.

### Stably transfected cell lines

Various constructs based on hGPR35a and mGPR35 were stably transfected into doxycycline inducible Flp-In TREx 293 cells. The pcDNA5/FRT/TO vector containing the relevant cDNA was transfected into cells using the FRT stable integration site. Cells were cotransfected with the relevant cDNA/pcDNA5/FRT/TO construct and a pOG44 Flp recombinase vector in a 1:8 ratio using PEI. After 48 h, cells were subcultured 1:10 and 1:30, and 24 h later, medium was changed to maintenance medium plus 200 μg/ml hygromycin to select for stable transfectants. Medium was changed every 3 days until individual colonies were visible by eye (10–14 days). Cells were then detached by incubating with trypsin-EDTA and pooled to give polyclonal cell lines, which were maintained in hygromycin selection medium. When required, expression of the integrated gene was induced by addition of 100 ng/ml doxycycline for 18 to 24 h.

### Cell lysate preparation

Cell lysates were generated from HEK293T cells following transient transfection to express receptor-HA fusion constructs. Cells were harvested in ice-cold PBS and lysed in lysis buffer (150 mM NaCl, 50 mM Tris–HCl, 5 mM EDTA, 1% Nonidet P-40, 0.5% Na-deoxycholate, and 0.1% SDS), supplemented with protease and phosphatase inhibitor tablets on a rotating wheel for 30 min at 4 °C. Samples were then centrifuged for 15 min at 11,000*g* at 4 °C. Protein content was assessed using a bicinchoninic acid protein assay kit (Thermo Fisher Scientific).

### Receptor immunoprecipitation and immunoblotting

HA-tagged receptor constructs were immunoprecipitated from 200 μl cell lysate using a monoclonal anti-HA antibody linked to agarose. Immunocomplexes were washed three times in wash buffer, resuspended in 100 μl Laemmli buffer, and incubated at 60 °C for 5 min. Following centrifugation at 2500*g* for 5 min, 20 μl of immunoprecipitated proteins were resolved by SDS-PAGE on NuPAGE Novex 4 to 12% Bis-Tris Gels (Thermo Fisher Scientific). Gels were run in NuPAGE MOPS SDS Running Buffer (Thermo Fisher Scientific) at 200 V for 50 min, and proteins were transferred from the gel onto nitrocellulose membrane using a wet transfer system. Following transfer, the nitrocellulose membrane was blocked using 5% bovine serum albumin (BSA) in Tris-buffered saline (TBS, 50 mM Tris-Cl, 150 mM NaCl, pH 7.6) for 1 h at room temperature on an orbital shaker. The membrane was then incubated with appropriate primary antibody in 5% BSA TBS supplemented with 0.1% Tween (TBS-T) overnight at 4 °C. Anti-pSer^300^/pSer^303^-hGPR35a and pSer^298^/pSer^301^-mGPR35 (5) were diluted 1:1000, and anti-HA was diluted 1:10,000. The membrane was washed (3 × 5 min with TBS-T) and incubated for 2 h at room temperature with IRDye 800CW anti-rabbit or anti-rat secondary antibody diluted 1:10,000 in 5% BSA TBST. After washing (3 × 5 min with TBS-T), proteins were detected using a LI-COR Odyssey imaging system according to the manufacturer’s instructions. For GRK isoform–directed antisera and the anti-LgBiT mAb, the following dilutions were used: Anti- LgBiT mAb: 1:500, anti-GRK 2 to 1:500, anti-GRK 3 to 1:250, anti-GRK 5 to 1:250, and anti-GRK 6 to 1:1000. IRDye 800CW anti-rabbit or anti-mouse secondary antibody diluted 1:10,000 in 5% dried Skimmed Milk Powder in TBST.

### Immunocytochemistry studies

Immunocytochemical experiment of the human and mouse GPR35 upon treatment with agonist ligands and GRK5/6 inhibitors were conducted according to previously described protocols (5).

### Ligand treatments

To assess agonist-dependent receptor phosphorylation, cells were serum starved for 1 h, then pretreated with vehicle or inhibitors for 15 min at 37 °C in a 5% CO_2_ humidified atmosphere. They were then exposed to agonist for a further 5 min. After treatment, cells were put on ice and harvested in ice-cold PBS.

### Arrestin-2/3 recruitment BRET assays

HEK293T cells were seeded in 10 cm^2^ dishes and transiently cotransfected with WT or mutant forms of orthologues of GPR35-eYFP, each with a FLAG epitope tag engineered into the N-terminal domain, and with arrestin-3 fused to *Renilla* luciferase (arrestin-3-RLuc) in a 4:1 ratio using PEI. Control cells were transfected with arrestin-3-RLuc only. After 24 h, cells were detached by incubating with trypsin-EDTA and seeded at 6 × 10^4^ cells/well in poly-D-lysine coated white 96-well plates, then incubated overnight at 37 °C. Cells were washed once with prewarmed (37 °C) Hanks’ buffered saline solution (HBSS) and incubated in HBSS for 30 to 60 min at 37 °C. During incubation, the eYFP signal (excitation 485 nm, emission 520 nm) was read on a PHERAstar FS (BMG Labtech) to estimate relative receptor expression. The RLuc substrate coelenterazine-h (Promega) was added to a final concentration of 5 μM, and the plate incubated for 10 min at 37 °C protected from light. Agonists were added at the relevant concentrations in triplicate, and the plate was incubated for a further 5 min at 37 °C, then the emissions at 475 nm and 535 nm were read on a PHERAstar FS. Net BRET values were obtained by dividing the emission at 535 nm by the emission at 475 nm and subtracting the 535 nm/475 nm ratio for cells expressing only the arrestin-3-RLuc donor (the basal BRET): Net BRET = (em535 nm/em475 nm) − (em535 nm/em475 nm [RLuc only]). Studies with arrestin-2-RLuc were performed in an equivalent manner.

### Receptor internalization studies

Flp-In-T-REx 293 cells stabling expressing hGPR35a-Nluc in a doxycycline-inducible manner were transiently transfected with 4.5 μg of Lyn11-mNG in pcDNA3.1 with a 1:5 ratio of DNA:PEI in T25 flasks. The following day cells were dissociated with TrypLE Ex and seeded into poly-D-lysine-coated white 96 well plates in DMEM (10% FBS, pen/strep, 100 ng/ml doxycycline) and incubated at 37 °C with 5% CO_2_ overnight. Cells were washed and medium replaced with HBSS (10 mM Hepes, pH 7.4). GRK inhibitors (10 μM) or vehicle was added to cells 1 h before, and coelenterazine-h (5 μM) 30 min before plates were read, lodoxamide (0.1 nM–1 μM) was then added, and plates were read every 2 min for an additional 40 min. Fold change of the pre-lodoxamide BRET ratio was calculated as was area under the curve. These values were then normalized to vehicle as 0% and lodoxamide 1 μM as 100%.

As an alternative method to assess receptor internalization we employed an assay that measures movement of proteins to early endosomes. Here Flp-In-T-REx 293 cells stably expressing hGPR35a-Nluc in a doxycycline-inducible manner were transiently transfected with 5 μg of the early endosome located marker mNG-FYVE in pcDNA3.1 with a 1:5 ratio of DNA:PEI. The following day cells were dissociated with TrypLE Ex and seeded into poly-D-lysine-coated white 96-well plates in DMEM (10% FBS, pen/strep, 100 ng/ml or 1.25 ng/ml doxycycline) and incubated at 37 °C with 5% CO_2_ overnight. Cells were washed and medium replaced with HBSS (10 mM Hepes, pH 7.4). Coelenterazine-h (5 μM), along with either GRK inhibitors (10 μM) or vehicle, was added to cells 15 to 30 min before plates were read, lodoxamide (0.1 nM–1 μM) was then added, and plates were read every 2 to 2.5 min for an additional 40 min. Fold change of the pre-lodoxamide BRET ratio was calculated at 30 min. These values were then normalized to vehicle as 0% and lodoxamide 1 μM as 100%.

### NanoBit GRK recruitment assay

HEK293T cells were transfected with 5 μg total DNA with 50 ng LgBit-GRK plasmid, 500 ng hGPR35a-SmBit plasmid, and 4450 ng of empty vector pcDNA3.1 using PEI at a 1:5 DNA:PEI ratio. Transfections were either directly into cells in poly-D-lysine coated white 96-well plates at time of seeding, or in 6 cm dishes and then seeded into dishes the following day at 40,000 cells per well. Cells were washed twice with HBSS (10 mM Hepes) buffer and then 70 μl buffer was added to each well. Substrate was added (10 μl per well, final 5 μM). GRK inhibitors or vehicle were added 15 min prior to agonist. Lodoxamide concentration-response curves were then calculated as fold change over their respective vehicle only values. GRK inhibitor concentration curves were calculated as fold change over the lodoxamide only curves.

### AlphaFold modeling

AlphaFold predictions (30, 38, 39) were made using the colabfold notebook (https://colab.research.google.com/github/sokrypton/ColabFold/blob/main/batch/AlphaFold2_batch.ipynb) using the human GPR35a sequence along with each of the GRK proteins, as well as arrestin-3, in multimer mode. Rank 1 models were superimposed in Mol∗ (https://molstar.org/viewer/) based on the predicted transmembrane helices of GPR35 (residues 17–44, 54–79, 87–119, 130–157, 173–198, 206–242, 249–276).

### Data analysis

All data are presented as means ± SD of at least three independent experiments. Data analysis and curve fitting was carried out using the GraphPad Prism software package version 8 (GraphPad; www.graphpad.com). For functional assays the concentration-response data were plotted on a log axis, with the untreated vehicle control plotted at 1 log unit lower than the lowest ligand concentration and fitted to a three-parameter sigmoidal curve with the Hill slope constrained to equal 1. To perform the statistical analysis of curve parameters, data from multiple experiments were fitted independently and resulting curve fit values were analyzed with indicated tests.

## Data availability

All data is freely available upon request to the corresponding author Graeme.milligan@glasgow.ac.uk.

## Supporting information

This article contains supporting information (14).

## Conflict of interest

The authors declare that they have no conflicts of interest with the contents of this article.

## Figures and Tables

**Supplemental Figure 1:**
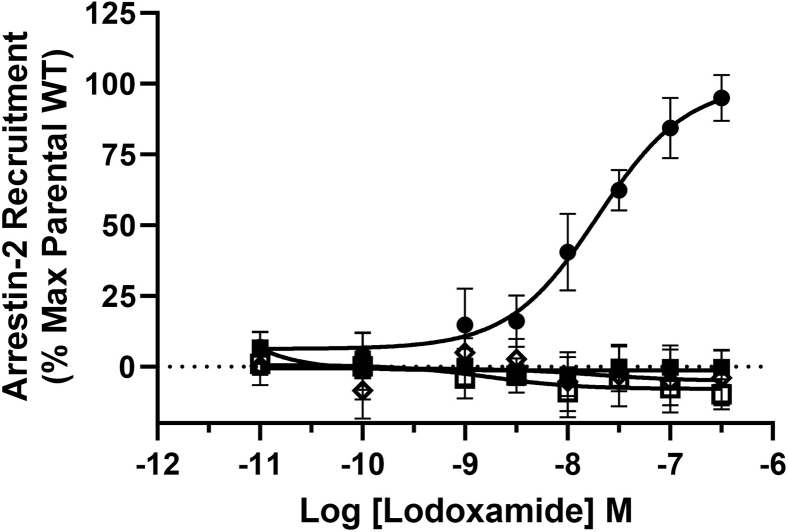
**Agonist-induced interactions between human GPR35a and arrestin-2 require the presence of one or more GRK isoform.** Parental HEK293 cells (*filled circles*, *open diamonds*) or a clone genome-edited to lack expression of each of GRK2, GRK3, GRK5 and GRK6 (ΔGRK2/3/5/6) (*filled squares*, *open squares*) were transfected transiently to express either hGPR35a-eYFP (*filled symbols*) or hGPR35a-PDM-eYFP (*open symbols*) and arrestin-2-RLuc. Cells were exposed to the indicated concentrations of lodoxamide for 5 minutes and after substrate addition BRET was measured. Data are the mean ± S.D. of outcomes from three independent experiments.

**Supplemental Figure 2:**
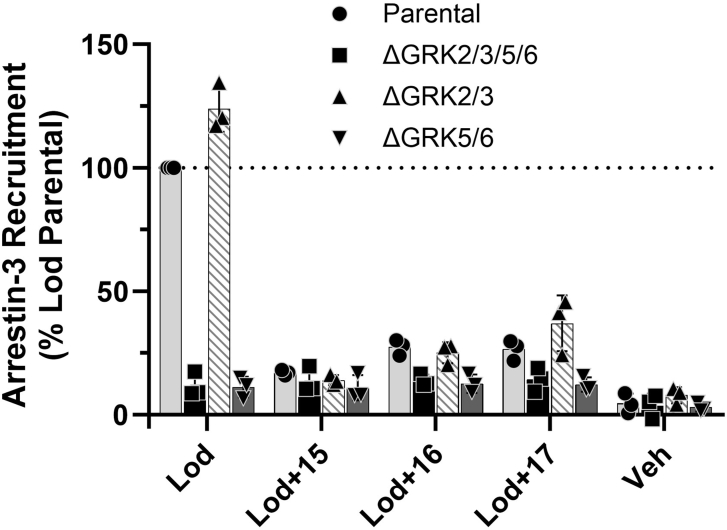
**A selection of compounds with GRK5/6 inhibitory activity prevent interactions between human GPR35a and arrestin-3.** Studies akin to Figure 1 were performed after transient co-expression of hGPR35a-eYFP and arrestin-3-RLuc into each of parental HEK293 cells (*circles*) or clones genome-edited to lack expression of GRK2 and GRK3 (ΔGRK2/3) (*triangles*), GRK5 and GRK6 (ΔGRK5/6) (*inverted triangles*) or all four of these (ΔGRK2/3/5/6) (*squares*). Cells were exposed to 100 nM lodoxamide for 5 min after pre-treatment with either vehicle or the indicated GRK5/6 inhibitor (compounds 15–17) (14) for 30 min. After substrate addition BRET was measured. Data are the mean ± S.D. of outcomes from three independent studies.

**Supplemental Figure 3A:**
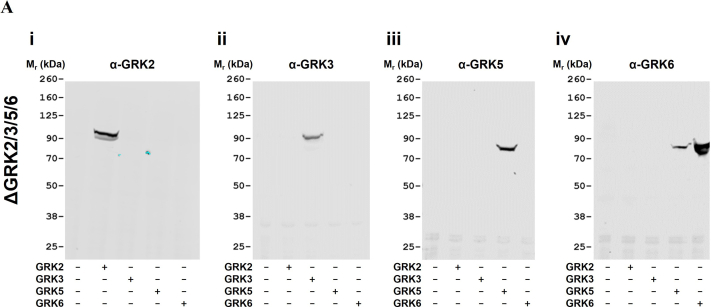
**Expression patterns of endogenous and transfected GRKs in HEK293-derived cell lines.** Following transfections akin to those in Figure 3, cell lysates were produced and resolved by SDS-PAGE. These (*A*, ΔGRK2/3/5/6 cells, *B*, parental HEK293 cells) were then immunoblotted with antibodies against GRK2 (**i**), GRK3 (**ii**), GRK5 (**iii**) and GRK6 (**iv**). In *B* relative levels of endogenous GRKs and the introduced LgBiT-tagged forms can be observed for each isoform because of the additional size (18 kDa) of the Lg-Bit fragment.

**Supplemental Figure 3B:**
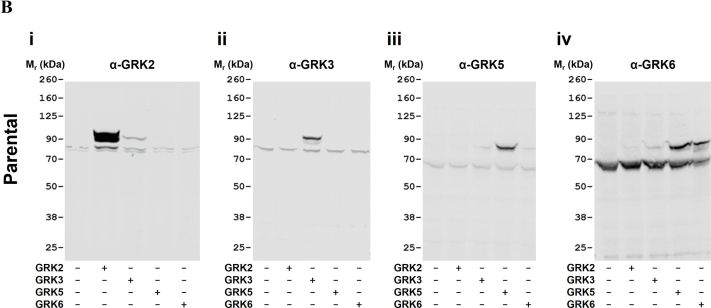


**Supplemental Figure 4:**
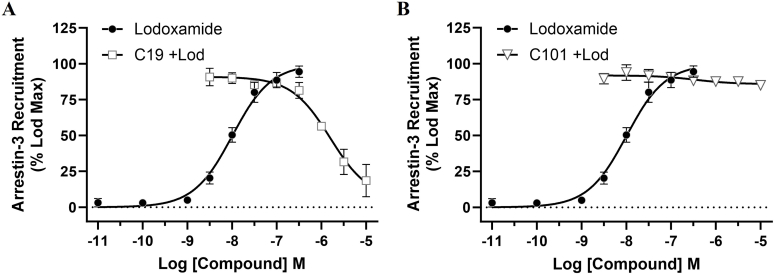
**Compound 19 prevents lodoxamide-induced hGPR35a-arrestin-3 interactions in parental 293 cells in a concentration-dependent manner.** Parental HEK293 cells were transfected transiently to express hGPR35a-eYFP and arr-3-RLuc. Prior to addition of lodoxamide (100 nM, 5 min) cells were pre-treated for 30 min with varying concentrations of either compound 19 (*A*) or compound 101 (*B*) (*open symbols*). Data are presented as % of the effect of lodoxamide in parental 293 cells in the absence of compound 19 or 101. A concentration-response curve of lodoxamide-induced hGPR35a-eYFP-arr-3-RLuc interactions in parental 293 cells (*filled circles*) is shown for reference. Data are the mean ± S.D. of outcomes from 3 independent experiments.
